# Epithelial Cell Damage Activates Bactericidal/Permeability Increasing-Protein (BPI) Expression in Intestinal Epithelium

**DOI:** 10.3389/fmicb.2017.01567

**Published:** 2017-08-15

**Authors:** Arjun Balakrishnan, Dipshikha Chakravortty

**Affiliations:** ^1^Department of Microbiology and Cell Biology, Indian Institute of Science Bangalore, India; ^2^Centre for Biosystems Science and Engineering, Indian Institute of Science Bangalore, India

**Keywords:** BPI, innate immunity, pore forming toxins, *S. aureus*, epithelial damage, p38 signaling, anti-microbial peptide

## Abstract

As the first line of defense against invading pathogen, intestinal epithelium produces various antimicrobial proteins (AMP) that help in clearance of pathogen. Bactericidal/permeability-increasing protein (BPI) is a 55 kDa AMP that is expressed in intestinal epithelium. Dysregulation of BPI in intestinal epithelium is associated with various inflammatory diseases like Crohn’s Disease, Ulcerative colitis, and Infectious enteritis’s. In this paper, we report a direct correlation between intestinal damage and BPI expression. In Caco-2 cells, we see a significant increase in BPI levels upon membrane damage mediated by *S. aureus* infection and pore-forming toxins (Streptolysin and Listeriolysin). Cells detect changes in potassium level as a Danger-associated molecular pattern associated with cell damage and induce BPI expression in a p38 dependent manner. These results are further supported by *in vivo* findings that the BPI expression in murine intestinal epithelium is induced upon infection with bacteria which cause intestinal damage (*Salmonella* Typhimurium and *Shigella flexneri*) whereas mutants that do not cause intestinal damage (STM Δ*fliC* and STM Δ*invC)* did not induce BPI expression. Our results suggest that epithelial damage associated with infection act as a signal to induce BPI expression.

## Introduction

The differentiation of self and non-self is an important aspect of both innate and adaptive immune response. Eukaryotic cells detect the presence of a pathogen by recognizing conserved microbial structures derived from pathogens collectively known as PAMPs (pathogen-associated molecular patterns). Detection of PAMPs by innate immune cells leads to the production of AMPs (anti-microbial peptides) and ROS (reactive oxygen species) that will help in clearance of pathogen. Bactericidal/permeability-increasing protein (BPI) is an anti-microbial protein which actively inhibits invasion by Gram-negative bacteria. The high affinity of BPI for LPS compared to its counterpart LBP (Lipopolysaccharide binding protein) makes BPI an anti-inflammatory molecule as well (Krasity et al., 2011). In humans, BPI is known to be present mostly in neutrophils (Weersink et al., 1993). Recent work by Colgan’s group showed expression of BPI in the intestinal epithelial cells. In intestinal epithelial cells, BPI expression is known to be regulated by anti-inflammatory lipid mediator ATLA4 (aspirin-triggered lipoxin A4) (Canny et al., 2002). Most cytokines and infection models have failed to induce expression of BPI in intestinal epithelial cells showing a tight control on BPI expression in these cells (Canny et al., 2006). Unlike immune cells, regulation of immune response in intestinal epithelium is very complex. Intestinal epithelial cells are constantly exposed to various PAMPs derived from Gut microbiota (Abreu, 2010). This constant exposure makes it difficult for intestinal epithelial cells to recognize PAMPs as a signature for a pathogen. The presence of BPI in gut epithelium helps us to use BPI as a model to understand the regulation of AMP expression in the gut epithelium.

In this study, we screened for various inflammatory signals for inducing BPI expression in intestinal epithelial cells. We found that pore-forming toxins induce BPI expression in intestinal epithelial cells. The cell damage associated changes in potassium levels act as Damage Associated Molecular Patterns (DAMPs) and induce BPI expression in a p38 dependent manner. As expected, none of the PAMPs screened induced BPI expression in epithelial cells. Interestingly, *Salmonella* Typhimurium (STM) which did not induce BPI expression *in vitro*, induced BPI expression in the murine intestinal epithelium *in vivo* which is the outcome of inflammation associated epithelial damage. Mutants of STM that cause less epithelial damage also showed less BPI expression. Together, these results indicate that intestinal epithelial cells recognize DAMPs as a signal for epithelial damage and induce BPI expression.

## Results

### *Staphylococcus aureus* Infection Induces BPI Expression in Human Intestinal Epithelial Cells

**FIGURE 1:**
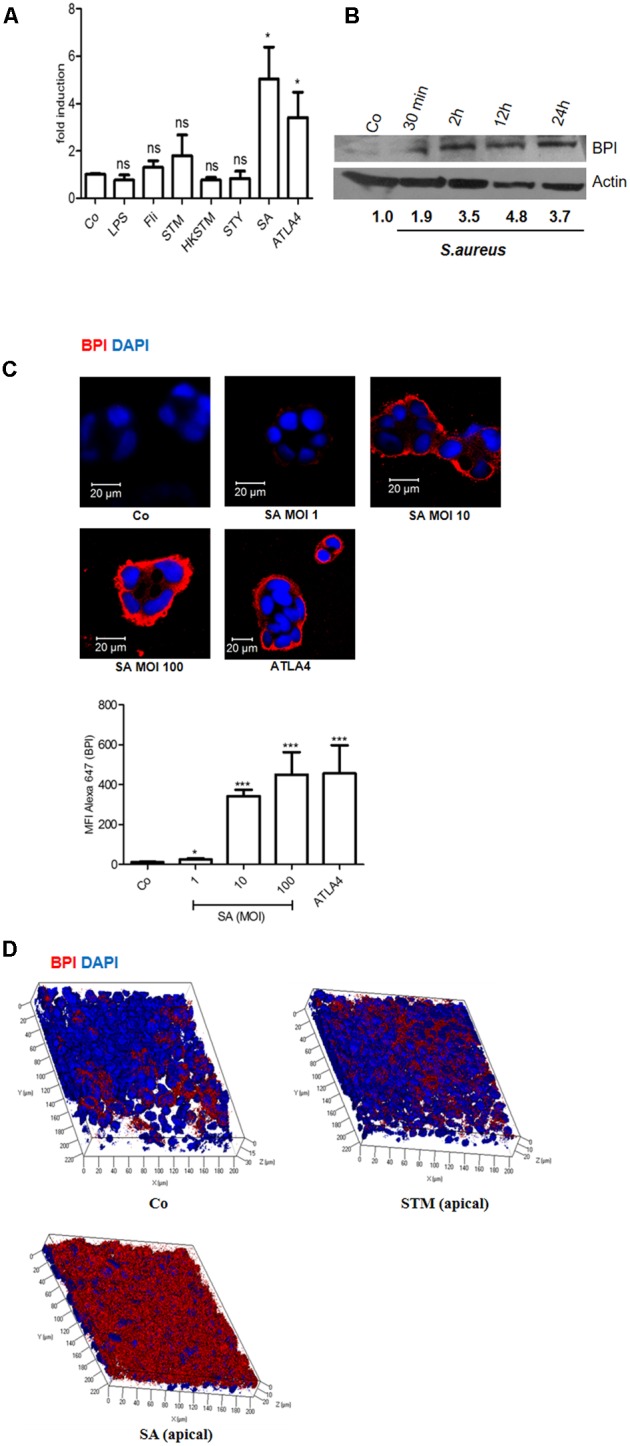
Bactericidal/permeability-increasing protein is induced in Caco-2 cells upon *Staphylococcus aureus* infection. Caco-2 cell monolayers were treated with LPS (100 ng/mL), Flagellin (500 ng/mL), *Salmonella* Typhimurium 14028 (STM, MOI 10), Heat Killed STM (HKSTM), *Salmonella* Typhi CT18 (STY, MOI 10), *S. aureus* 25923 (SA MOI 10), or ATLA4 (aspirin-triggered lipoxin A4). **(A)** Total RNA was isolated 24 h post-treatment and BPI levels were determined using real-time PCR. (*n* = 5 experiments). Statistical analysis was done by the students’ *t*-test. **(B)** Western Blot showing BPI expression following SA infection at indicated time points. BPI levels were normalized to β actin internal control and expressed relative to medium alone control (Co). (*n* = 3 experiments). **(C)** Immunostaining showing BPI expression in Caco-2 cells post-infection with indicated MOI of SA. ATLA was used as positive control. Bottom: The Mean Fluorescent Intensity (MFI) of BPI was calculated using Zen software and plotted. **(D)** Caco-2 cells were seeded in 0.45 μ tissue culture inserts and were allowed to polarize for 8 days, polarized cells were infected with STM or SA and BPI expression was analyzed using Immunostaining. For **C** and **D**, Cells were stained with anti-BPI antibody followed by anti-antibody conjugated with Alexa 647 (red). Nuclei were labelled with 4’, 6-diamidino-2-phenylindole (DAPI; blue). Cells were imaged by confocal microscopy. Representative images are shown. (*n* = 4 experiments). Key: ^∗∗∗^*p* < 0.001, ^∗∗^*p* < 0.005, ^∗^*p* < 0.05, ns = not significant.

To explore the link between infection and BPI expression in intestinal epithelial cells, we analyzed the expression of BPI in Caco-2 cells upon bacterial infection. Caco-2 cells were infected with different pathogens viz STM, *Salmonella* Typhi (STY), and *Staphylococcus aureus* (SA) at a multiplicity of infection (MOI) of 10. Twenty-four hours post-infection, RNA was isolated and BPI expression was quantified by real-time PCR. ATLA4 (aspirin-triggered lipoxin A4) was used as a positive control in the experiment (**Figure 1A**). Interestingly, BPI expression increased up to fivefold upon SA infection compared to uninfected control. As expected, BPI expression increased up to threefold upon ATLA4 treatment. Infection with STM, STY or treatment with different Pathogen Associated Molecular Patterns (PAMPs) viz LPS (100 ng), Flagellin (500 ng) and Heat Killed STM (HK STM) did not significantly influence BPI expression in Caco-2 cells.

In order to evaluate BPI expression at protein level, Caco-2 cells were infected with *S. aureus* at an MOI of 10. Cells were lysed at indicated time points (30 min, 2, 12, and 24 h), total protein was isolated and BPI expression was checked by western blotting (**Figure 1B**). BPI expression significantly increased in a time-dependent manner in SA infected cells compared to uninfected control. There was up to fourfold increase in BPI expression within 24 h post-SA infection compared to uninfected control. SA infection induced BPI expression in HeLa cells as well, indicating a common mode of regulation in these cells (Supplementary Figure S1). To understand the correlation between bacterial load and BPI expression, we checked BPI levels in Caco-2 cells upon infection with different MOI of SA (1, 10, or 100). Twenty-four hours post-infection, cells were fixed and BPI expression was checked by confocal microscopy (**Figure 1C**). BPI expression increased in an MOI-dependent manner in Caco-2 cells as analyzed by quantifying the MFI (Mean-fluorescent intensity) of BPI expression. Maximum expression of BPI was seen at MOI of 100. ATLA4 was used as a positive control in the experiment (Canny et al., 2006). These results suggest that BPI expression in epithelial cells is induced upon SA infection in an MOI and time-dependent manner. To further confirm these results, we checked BPI expression in polarized epithelial cells. Caco-2 cells were seeded in 0.4 μ tissue culture inserts. Eight days post-seeding, cells were infected with STM and SA at an MOI of 10. Cells were fixed and BPI expression was analyzed by confocal microscopy (**Figure 1D**). BPI expression was significantly high in polarized Caco-2 cells upon *S. aureus* infection compared to uninfected control. BPI expression remained unchanged upon STM infection. These results confirm that BPI levels are increased in intestinal epithelial cells upon SA infection.

### Epithelial Cells Respond to Cell Damage and Induce BPI Expression in a p38 Dependent Manner

**FIGURE 2:**
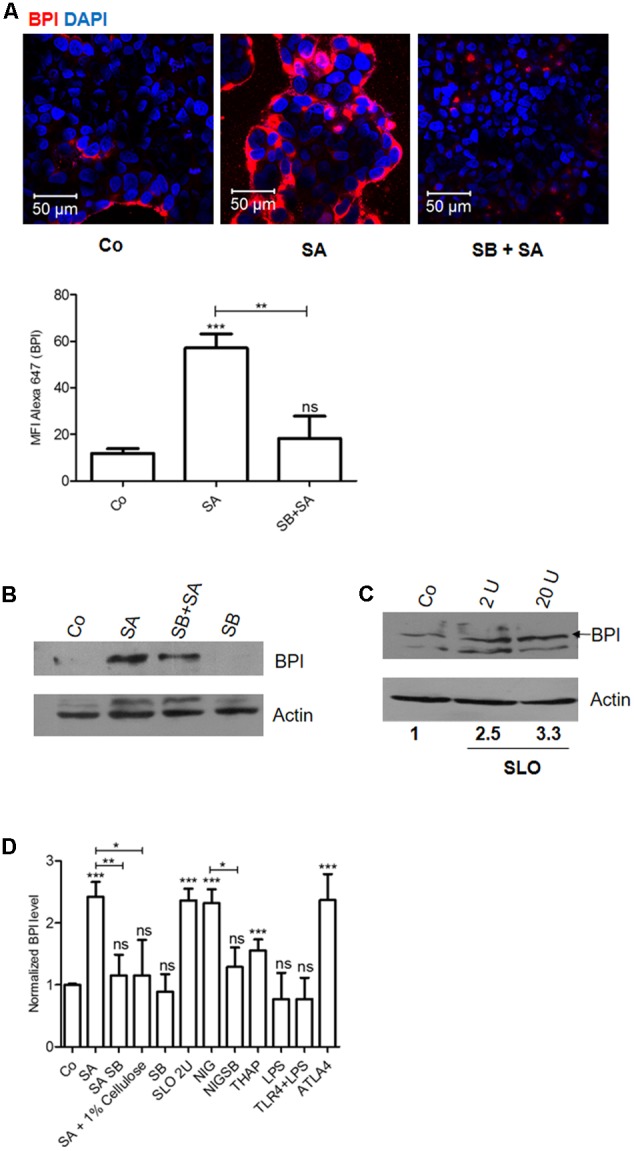
Caco-2 cells recognize *Staphylococcus aureus* induced stress and increase BPI expression in a p38 dependent manner. Caco-2 cell monolayers were either left untreated or treated with SB203580 (p38-MAPK inhibitor, SB) 1 h before *S. aureus* (SA) infection. **(A)** Cells were fixed 24 h post-infection and were immunostained with anti-BPI antibody. Representative images are shown. Bottom: The Mean Fluorescent Intensity (MFI) of BPI was calculated using Zen software and plotted. **(B)** Cells were lysed 24 h post-infection and BPI levels were checked by western blot. (*n* = 4 experiments) Co (media alone). **(C)** Cells were treated with Streptolysin O (2, 20 units) for 30 min and BPI levels were checked by western blot 24 h post-treatment. (*n* = 3 experiments). **(D)** BPI protein levels in Caco-2 cells were normalized to β actin internal control and expressed relative to medium alone control. The data is expressed as relative BPI levels (*x*-axis; *n* = 4 experiments). Statistical analysis was done by the students’ *t*-test. Key: ^∗∗∗^*p* < 0.001, ^∗∗^*p* < 0.005, ^∗^*p* < 0.05, ns = not significant.

*Staphylococcus aureus* is known to produce pore-forming toxins that can damage the epithelial cells (Ratner et al., 2006). Epithelial cells respond to these cellular stresses through activation of MAPK signaling pathways (Huffman et al., 2004; Husmann et al., 2006; Aroian and van der Goot, 2007). We hypothesized that BPI expression induced by SA infection might be mediated through p38 MAPK signaling pathway. To check this hypothesis, we pretreated Caco-2 cells with p38 MAPK inhibitor (SB203580) and infected cells with SA. Twenty-four hours post-infection, cells were lysed and BPI expression was quantified by western blot (**Figures 2B,D**). Pretreatment of Caco-2 cells with SB203580 significantly reduced SA induced BPI expression. To confirm this result, we checked the expression of BPI under similar conditions using confocal microscopy. Cells pretreated with p38 MAPK inhibitor were infected with SA. Twenty-four hours post-infection, cells were fixed and BPI expression was analyzed by using confocal microscopy (**Figure 2A**). Pretreatment of cells with SB203580 significantly reduced SA induced BPI expression as quantified by evaluating the MFI of BPI expression. These results suggest that SA induced BPI expression is in a p38 dependent manner.

To understand whether SA induced BPI expression is a response to pore formation in infected cells, we checked BPI expression in Caco-2 cells after treating the cells with pore-forming toxins. Caco-2 cells were treated with SLO (Streptolysin O) for 24 h. Cells were lysed and BPI expression was quantified by western blotting (**Figures 2C,D**). SLO treatment increased BPI expression up to 2.5-fold compared to untreated control. This increased BPI expression in epithelial cells in a concentration-dependent manner further confirmed the specificity of induction (**Figure 2C**). To supplement these results, we quantified BPI expression in Caco-2 cells after treating the cells with Listeriolysin O (LLO). Twenty-four hours post-treatment, BPI expression was analyzed by confocal microscopy and western blotting (Supplementary Figures S2A,B). LLO treated Caco-2 cells showed increased expression of BPI compared to untreated control. These results confirm that pore-forming toxins induce BPI expression in epithelial cells.

### Cells Perceive Changes in Potassium Level as a DAMP (Danger Associated Molecular Pattern) and Induce BPI Expression in a p38 Dependent Manner

**FIGURE 3:**
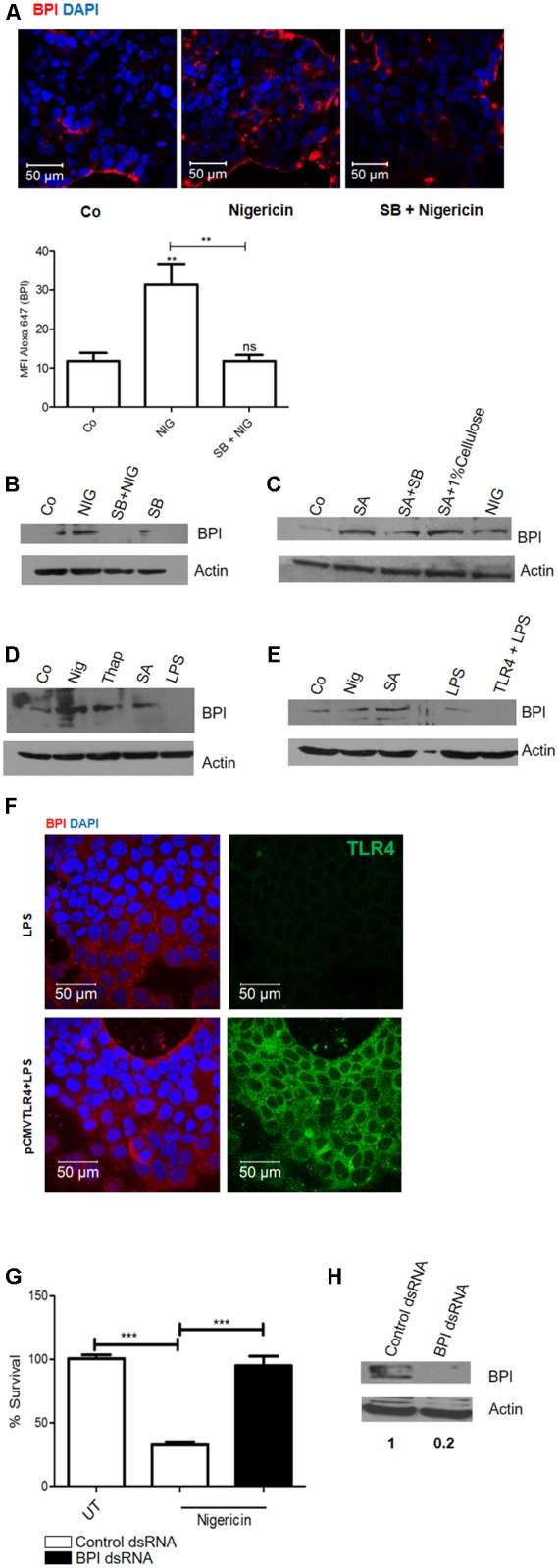
Epithelial cells perceive changes in potassium level as a DAMP (Danger Associated Molecular Pattern) and induce BPI expression in a p38 dependent manner. Caco-2 cells were treated with nigericin (6 μm) in presence or absence of p38 inhibitor (SB203580). **(A)** Cells were fixed 24 h post-treatment and BPI levels were visualized by immunostaining using *(anti-BPI antibody. Representative images are shown. Bottom: The Mean Fluorescent Intensity (MFI) of BPI was calculated using Zen software and plotted. **(B)** Cells were lysed and BPI levels were quantified using western blot. Co (DMSO control). (*n* = 4 experiments). **(C)** Cells were treated with 1% cellulose along with SA infection and BPI levels were checked by western blot. (*n* = 3 experiments). **(D)** Cells were treated with Thapsigargin (500 nm), Nigericin (6 μm), SA (MOI 10) or LPS (100 ng/mL). Twenty-four hours post-treatment, cells were lysed and BPI levels were quantified using western blot. (*n* = 4 experiments). **(E)** Caco-2 cells transfected with huTLR4 was exposed to LPS for 24 h. Total protein was isolated and BPI levels were quantified by western blotting. Nigericin treatment and SA infection was used as positive control. (*n* = 3 experiments). **(F)** Cells were fixed and BPI levels were visualized by immunostaining using anti-BPI antibody. Flag tagged TLR4 was visualized using anti-TLR4 antibody. Representative images are shown. **(G)** Cells were transfected with BPI dsRNA or scrambled control dsRNA, 24 h post-transfection cells were treated with nigericin. Twenty-four hours post-treatment, cells were infected with *Salmonella* Typhimurium and bacterial survival was quantified 2 h post-infection (*n* = 3 experiments). Statistical analysis was done by the students’ *t*-test. **(H)** Western Blot showing BPI expression following BPI dsRNA transfection in Caco-2 cells. Key: ^∗∗∗^*p* < 0.001, ^∗∗^*p* < 0.005, ns = not significant.)*

Three major and immediate changes associated with pore formation which induces a survival signal in epithelial cells are (a) decrease in cellular potassium levels, (b) increase in cellular calcium levels, and (c) osmotic pressure (Aroian and van der Goot, 2007). We hypothesized that change in potassium levels might act as a DAMP which can induce BPI expression in epithelial cells. To check this hypothesis, we treated Caco-2 cells with K^+^ ionophore (Nigericin). Twenty-four hours post-treatment, cells were fixed and BPI expression was analyzed by confocal microscopy (**Figure 3A**). Nigericin treatment led to significant upregulation of BPI expression compared to control as quantified by increase in MFI of BPI in nigericin treated cells (**Figure 3A**). To understand the role of p38 MAPK signaling pathways in inducing nigericin mediated BPI expression, Caco-2 cells were pretreated with p38 MAPK inhibitor, and BPI expression was analyzed following nigericin treatment (**Figure 3A**). Confocal analysis showed that pretreatment of cells with p38 MAPK inhibitor significantly affected nigericin induced BPI expression. To validate these results, BPI expression in Caco-2 cells post-nigericin treatment was quantified by western blotting (**Figures 2D, 3B**). Nigericin treatment led to a 2.5-fold increase in BPI expression compared to untreated control. Pretreatment of cells with p38 MAPK inhibitor decreased nigericin induced BPI levels (**Figures 2D, 3B**). Analysis of BPI expression in HeLa cells by western blotting revealed that Nigericin induces BPI expression in a p38 dependent manner (Supplementary Figure S3A). To check the importance of changes in osmotic pressure associated with cell damage in inducing BPI expression, Caco-2 cells were treated with an osmoprotectant (1% cellulose) along with SA infection and BPI levels were quantified by western blotting (**Figures 2D, 3C**). Treatment of Caco-2 cells with 1% cellulose couldn’t completely inhibit BPI expression mediated by SA infection. This indicates that other stress signals along with osmotic stress contribute to BPI expression in the intestinal epithelium.

Next, to check the importance of calcium influx in inducing BPI expression, Caco-2 cells were treated with Thapsigargin (calcium ionophore). This lead to a 1.6-fold increase in BPI expression compared to untreated control even though maximum induction of BPI expression was mediated by nigericin (**Figures 2D, 3D**). However, treatment of Caco-2 cells with LPS under similar conditions did not induce BPI expression (**Figures 2D, 3D**). Interestingly, most of the PAMPs tested can induce p38 phosphorylation in Caco-2 cells, proposing involvement of p38 independent signaling pathway in inducing BPI expression (Supplementary Figure S3B).

Previous studies have reported that epithelial cells have decreased expression of TLR4 (Abreu et al., 2001). To understand whether the inability of PAMPs to induce BPI expression in epithelial cells is due to the decreased expression of their cognate receptors, we overexpressed TLR4 in epithelial cells and tried to check BPI expression in epithelial cells after LPS treatment. Caco-2 cells were transfected with pcDNA TLR4, 24 h post-transfection, cells were treated with LPS. Cells were lysed and BPI expression was quantified by western blotting (**Figure 3E**). Transfected cells were examined by confocal microscopy for expression of membrane FLAG-tagged human TLR4 (**Figure 3F**). LPS treatment, following TLR4 expression, led to increase in IL-8 mRNA levels as quantified by real time PCR (Supplementary Figure S5). But under similar conditions, there was no significant increase in BPI expression in transfected cells compared to control (**Figure 3E**). These results prove that BPI expression in epithelial cells might be exclusively mediated through DAMP signals.

To understand whether DAMP associated BPI induction can contribute to bacterial clearance, nigericin treated Caco-2 cells were infected with STM at an MOI of 10. Bacterial numbers in Caco-2 cells were quantified by plating the cell lysates 2 h post-infection. Interestingly, treatment with nigericin led to a 65% decrease in bacterial survival compared to untreated control (**Figure 3G**). To check the contribution of BPI in limiting bacterial survival under these conditions, bacterial survival was quantified upon BPI knock down in Caco-2 cells. Efficacy of BPI knockdown in dsRNA transfected cells was validated by western blot analysis (**Figure 3H**). Knocking down of BPI in Caco-2 cells inhibited nigericin mediated bacterial killing confirming the role of BPI in bacterial killing. This result confirms that DAMP mediated BPI induction contribute to bacterial clearance.

### *In Vivo* Assessment of BPI Expression in Intestinal Epithelial Cells

**FIGURE 4:**
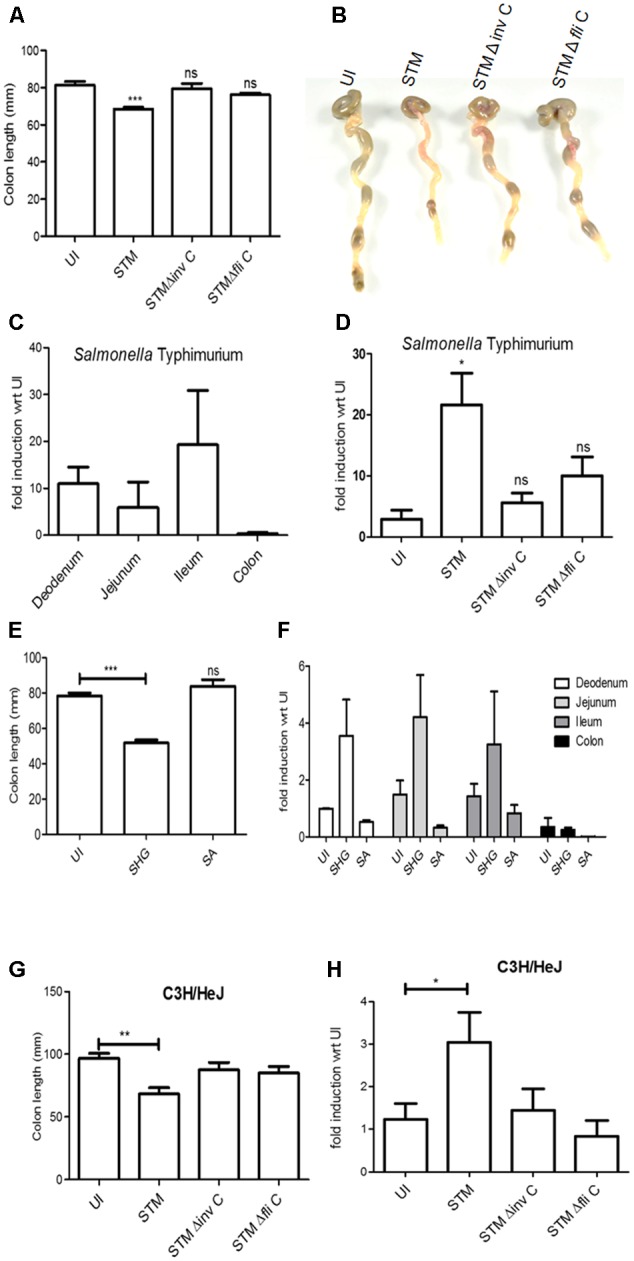
Bactericidal/permeability-increasing protein mRNA levels in mice intestine increases upon intestinal damage. BALB/c mice were infected intragastrically with 10^8^ bacteria (STM, STM Δ*fliC*, STM Δ*invC*). Two days post-infection mice were euthanized, intestine was isolated. **(A)** Colon length of mice post-infection (*n* = 9mice/group) **(B)** Representative images of colon post-bacterial infection. **(C)** real time-PCR analysis of BPI expression in different regions of intestine (Duodenum, Jejunum, Ileum, and Colon). Expression levels were normalized with same regions isolated from uninfected mice (placebo control). Statistical analysis was done by the students’ *t*-test. (*n* = 5mice/group). **(D)** Real-time PCR analysis of BPI expression in ileum post-bacterial infection. Expression levels were normalized with that of uninfected mice (placebo control). Statistical analysis was done by the students’ *t*-test. (*n* = 9mice/group). **(E)** BALB/c mice were infected intraperitoneally with 10^8^ bacteria (SHG) or intragastrically with 10^8^ bacteria (SA). One day post-infection mice were euthanized, intestine was isolated and colon length was measured (*n* = 5mice/group). **(F)** Real-time PCR analysis of BPI expression in ileum of mice infected as explained in **Figure 3E**. *(Expression levels were normalized with that of uninfected mice (placebo control). Statistical analysis was done by the students’ *t*-test. (*n* = 5mice/group). **(G)** C3H/Hej mice was infected with indicated bacteria. Two days post-infection mice were euthanized, intestine was isolated and colon length was measured (*n* = 5mice/group). **(H)** Real-time PCRS analysis of BPI expression in ileum of mice infected as explained in **Figure 3G**. Expression levels were normalized with that of uninfected mice (placebo control). Statistical analysis was done by the students’ *t*-test with respect to placebo control. (*n* = 5mice/group). *N* = 1 experiment. Key: ^∗∗∗^*p* < 0.001, ^∗∗^*p* < 0.005, ^∗^*p* < 0.05.)*

In order to understand the *in vivo* regulation of BPI expression during the course of bacterial infection, we checked the expression of BPI in the murine intestinal epithelium. Unlike *in vitro* conditions, infection by enteric pathogens is known to induce intestinal damage *in vivo*. To understand the regulation of BPI *in vivo*, mice were infected intragastrically with 1 × 10^8^ CFU of STM. Two days post-infection, mice were euthanized and intestine was removed for analysis. As expected, STM infection led to intestinal damage as seen by decreased colon length and increased histopathological score compared to uninfected mice (placebo control) (**Figures 4A,B** and Supplementary Figure S6). To quantify BPI expression under these conditions, total RNA was isolated from intestine and BPI expression was quantified by real-time PCR. STM infection led to a significant increase in BPI expression in various regions of the intestine viz duodenum, jejunum, ileum, and colon compared to uninfected mice (placebo control) (**Figure 4C**). Interestingly, maximum change in BPI expression was observed in the ileum (20-fold) which is known to be a preferential entry point for STM (Carter and Collins, 1974). To confirm the same, BPI expression was quantified in the mouse model of shigellosis (Yang et al., 2014). Mice were infected intraperitoneally with 10^8^ CFU of *Shigella flexneri* and BPI expression was quantified in various regions of the intestine, 1-day post-infection. *S. flexneri* infection led to severe damage in the intestine as seen by decreased colon length and increased histopatholological score compared to control mice (**Figure 4E** and Supplementary Figure S6). Under these conditions, we saw a significant increase in BPI expression in the duodenum, jejunum, and ileum compared to uninfected control (placebo control) (**Figure 4F**). Conversely, there was no increase in BPI expression during SA infection (1 × 10^8^ CFU intragastrically) and SA infection did not cause any damage to mice intestine (**Figures 4E,F** and Supplementary Figure S6). This points to a direct correlation between intestinal damage and BPI expression.

### BPI Expression in Murine Epithelial Cells Is Mediated by an Active Inflammatory Response

Intestinal damage during bacterial infection is an outcome of active inflammatory response against the invading pathogen (Abreu, 2010; Asquith et al., 2010; Fournier and Parkos, 2012). To comprehend the role of an active bacterial invasion and inflammatory response in inducing BPI expression, we checked BPI expression in mice after infection with an invasion deficient mutant STMΔ*invC* (Bueno et al., 2010). As expected, STMΔ*invC* showed decreased invasion of intestinal epithelial cells as seen by both reduced entry in Caco-2 cells and reduced bacterial burden in intestine compared to wild-type bacteria (Supplementary Figures S4A,B). There was no significant difference in the colon length or intestinal damage in STMΔ*invC* infected mice as compared to that of uninfected control (placebo control) (**Figures 4A,B** and Supplementary Figure S6). Additionally, there was no significant increase in BPI expression in STMΔ*invC* infected mice compared to uninfected control (placebo control) (**Figure 4D**).

Two major PAMPs which can mediate an active inflammatory response during the course of Salmonella infection are LPS and flagellin (Gewirtz et al., 2001; Roy et al., 2006; Santos et al., 2009). To understand the contribution of these PAMPs in inducing intestinal damage and BPI expression in the murine intestine, we checked BPI expression in two conditions. First, we checked BPI expression in the murine intestine after infecting with STMΔ*fliC*. Infection with STMΔ*fliC* showed less intestinal damage compared to STM wild type infected mice (**Figures 4A,B** and Supplementary Figure S6). Also, we couldn’t detect any significant increase in BPI expression in murine ileum as compared to that of uninfected mice (placebo control) (**Figure 4D**). This result suggests that inflammation associated intestinal damage act as a queue to induce BPI expression in the intestine. Next, we checked BPI expression in TLR4 knockout mice after infection with STM (WT) bacteria. STM infection in TLR4 knockout mice lead to severe damage in the intestine as seen by a significant decrease in colon length (**Figure 4G**). Interestingly, infection with STM WT in these mice showed two to threefold increase in BPI expression compared to uninfected control (placebo control) (**Figure 4H**). This increase in BPI level is a direct effect of epithelial damage. We couldn’t detect any change in BPI expression upon infection with STMΔ*invC* or STMΔ*fliC* in these mice compared to untreated control (**Figure 4H**). These results supports the notion that inflammation and associated damage induce BPI expression in murine intestinal epithelium.

## Discussion

**FIGURE 5:**
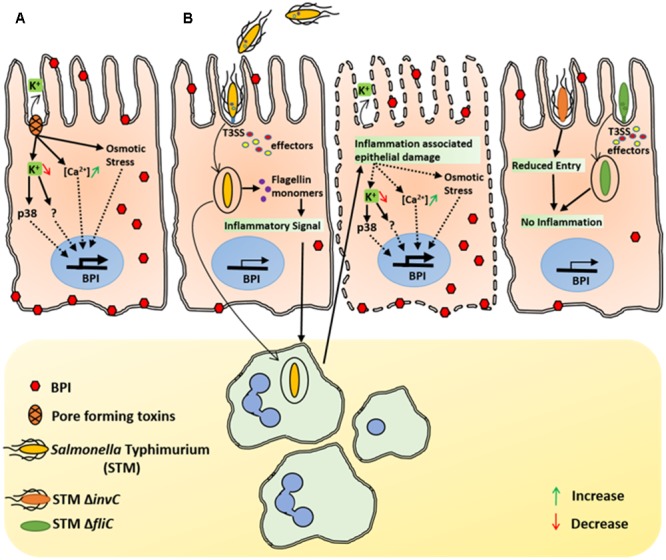
Inflammation associated epithelial damage induce BPI expression. **(A)** Under *in vitro* conditions, SA associated pore forming toxins mediates K^+^ efflux in Caco-2 cells. Epithelial cells detect changes in potassium levels and induce BPI expression in a p38 dependent manner. Increased Ca ^2+^ levels as well as osmotic stress associated with epithelial damage can also induce BPI expression in epithelial cells. **(B)** *In vivo*, STM induces bacterial uptake by epithelial cells in a SPI1 dependent manner. Internalized STM translocate flagellin in to the host cytosol and induces inflammatory signal that leads to recruitment of immune cells to the site of infection. Immune cells further elevate the inflammatory response, which leads to epithelial damage and induces BPI expression in intestinal epithelium. STM SPI1mutant that cannot invade intestinal epithelium (STM *invC*) as well as STM mutant which cannot provoke immune response (STM Δ*fliC*) will have diminished epithelial damage as well as less BPI expression.

“How a pathogen is recognized by the host?” is a fundamental question in immunology. It is relatively easy to answer this question in a sterile environment like blood or internal organs. The recognition of PAMPs in these environments helps to mount a proper immune response. But in a complex environment like intestine which is constantly exposed to gut micro flora and various metabolites and components derived from microbiota, the answer for the same question is also quite complex. In such an environment, it is difficult to distinguish between patterns derived from gut microbiota and PAMPs derived from infecting bacteria. Understanding the regulation of various immune modulators in the gut is important for designing various therapeutic intervenes against inflammatory disorders like Inflammatory Bowel Disease (IBD), Crohn’s Disease (CD), etc. In the present study, we tried to understand a major immune modulator in the intestine, BPI. BPI acts as both anti-bacterial and anti-inflammatory agent in the intestine. Previous studies have tried to screen various inflammatory agents to study the regulation of BPI in the intestine, but none of the inflammatory signals including PAMPs and cytokines could induce BPI expression in the intestine (Canny et al., 2006). We screened for various bacteria to induce BPI expression in the intestine. Of all the pathogens screened, SA was able to induce BPI expression in Caco-2 cells (**Figure 1A**). SA is known to produce pore-forming toxins which can target the infected cell membrane. Treatment of Caco-2 cells with pore forming toxins also lead to BPI induction in these cells. Production of BPI during pore formation might have a huge impact in regulating the microbiota-derived LPS mediated inflammatory response during epithelial damage. BPI expression associated with cell damage might restrict entry of normal microbiota during epithelial damage. Pore formation in intestinal epithelial cells leads to the alteration in cytosolic ion levels and osmotic pressure of these cells (Aroian and van der Goot, 2007). We tried to address the role of these changes in increasing BPI levels associated with cell damage. Our studies showed that changes in potassium levels act as a major signal in inducing BPI expression associated with pore formation (**Figure 5A**). Additionally, changes in Ca^2+^ levels and osmotic stress also contributed toward BPI expression to a certain extent. TLR4 overexpression and LPS treatment couldn’t induce BPI expression in Caco-2 cells (**Figure 3E**) indicating that rather than PAMPs, DAMPs signal the presence of the pathogen in the gut and induce BPI expression in intestinal epithelial cells. Interestingly, inhibition of p38 MAPK (a known sensor of stress associated signals) inhibited SA and Nigericin mediated BPI expression in Caco-2 cells. But, p38 is not the sole signaling pathway that leads to BPI expression in Caco-2 cells as most of the PAMPs tested can mediate p38 phosphorylation in the epithelial cell. Even though *Salmonella* did not induce epithelial damage in cell culture model, *Salmonella* infection in the mouse led to epithelial damage (Santos et al., 2009). This damage is due to an active immune response against the invading pathogen. Under resting conditions, maximum expression of BPI is seen in the duodenum and minimal expression of BPI is seen in the colon (Canny et al., 2006). These levels can be directly correlated to the number of bacteria in the gut microbiota, where maximum bacterial numbers are seen in the colon and minimum in the duodenum. Salmonella is known to invade primarily through distal ileum of the intestine (Carter and Collins, 1974). Interestingly, we see a maximum increase in BPI expression in the distal ileum compared to the duodenum, jejunum, and colon in comparison with uninfected control (**Figure 4C**). These results suggest that, under resting conditions, BPI expression is tightly regulated in the distal regions of the intestine. But, during the course of infection, maximum BPI expression is seen in the distal regions of the intestine, where there is a higher chance of infiltration by gut microbiota. BPI expression was analyzed by extracting total RNA from the intestine. Since BPI is exclusively expressed in cells derived from myeloid lineage and BPI mRNA is absent in both murine neutrophils and macrophages, we believe that most changes associated with BPI mRNA are contributed by intestinal epithelial cells (Balakrishnan et al., 2013). STMΔ*invC* (invasion deficient mutant) infection in mice couldn’t induce BPI expression in the ileum. STMΔ*invC* mutants have a defective secretion of SPI1 effectors which are important in mediating bacterial entry inside epithelial cells (Bueno et al., 2010). Since the bacterial entry was less, epithelial damage and inflammation was also less in mice infected with STMΔ*invC*. To understand the importance of inflammation in inducing BPI expression in the intestinal epithelium, mice were infected with STMΔ*fliC*. Flagellin is the major PAMP associated with intestinal inflammation during salmonella infection (Winter et al., 2009; Carvalho et al., 2012). Mice infected with STMΔ*fliC* showed reduced intestinal damage and BPI expression as well. These results point out a direct role of inflammation and associated intestinal damage in inducing BPI expression in the intestinal epithelium (**Figure 5B**). Further, STM infection in TLR4-/- mice displayed a significant increase in BPI expression compared to uninfected mice, but with respect to WT mice, STM mediated BPI expression is less in TLR4-/- mice. Even though TLR4 is a major sensor that detects LPS in Gram-negative bacteria, TLR4-/- mice shows increased intestinal damage compared to WT mice during STM infection (Fukata et al., 2005). TLR4 mediated signaling is important in restricting the growth of bacteria in the gut, even though the precise mechanism for the same is not clear (Vazquez-Torres et al., 2004). Increased intestinal damage in TLR4-/- mice is due to increased proliferation of *Salmonella* in the intestine (O’Brien et al., 1980). STMΔ*invC* and STMΔ*fliC* did not induce epithelial damage in TLR4-/- mice. These results suggest that STM induced inflammation in TLR4-/- mice is mediated by flagellin, further confirming the role of flagellin in Salmonella pathogenesis (Fournier et al., 2009). *In vitro* studies on BPI expression proves that pore formation in epithelium will lead to potassium-mediated activation of p38 signaling pathways culminating in BPI expression. Whether similar signaling pathways exist *in vivo* is not clear. Our results on BPI expression *in vivo* show that there is a direct link between epithelial damage and BPI expression. Along with *in vitro* data, these results lead us to hypothesize that similar signaling pathways exist *in vivo* as well. Intestinal inflammation also leads to recruitment of neutrophils to the site of injury (Canny and Levy, 2008). We cannot completely rule out the contribution of lipoxin signaling in inducing BPI expression under these conditions (Canny and Levy, 2008). Even though SA can infect intestinal epithelial cells, under *in vivo* conditions we could not detect any bacteria inside the intestine (data not shown). There was no significant inflammation associated with SA infection. Under these conditions, there was no increase in BPI levels as well. Previous reports show an increase in BPI expression in Canines upon infection with *E. coli* (Hagman et al., 2009). Interestingly, these *E. coli* strains had hemolytic properties. In this study, we see an increase in BPI levels in HeLa cells upon SA infection as well as Nigericin treatment (Supplementary Figures S1, S3). These results demonstrate that similar signaling pathways might exist in both intestine and cervix. As SA is one of the pathogens that causes cervicitis, SA-mediated BPI induction might have further implications under conditions where multiple pathogens are involved in infection (Muder et al., 2006). Studies which tried to correlate between BPI expression and intestinal inflammation shows a significant increase in BPI expression during ulcerative colitis (UC) (Haapamaki et al., 1999). Our study opens a new direction to this question suggesting the contribution of increased BPI expression in gut mucosa associated with intestinal damage. Further clinical studies have to be done to understand the importance of gut BPI in diseases associated with gut inflammation.

## Materials and Methods

### Ethics Statement

The animal experiments were carried out in accordance with the approved guidelines of institutional animal ethics committee at Indian Institute of Science, Bangalore, India (Registration No: 48/1999/CPCSEA). All procedures with animals were carried out in accordance with the institutional rules for animal experimentation under strict vigilance.

### Bacterial Strains

*Salmonella* Typhimurium (*Salmonella* enerica serovar Typhimurium ATCC14028s), STM Δ*invC*, STM Δ*fliC* (Flagellin-deficient STM 14028), STY (STY ATCC CT18), SHG (*S. flexneri* clinical isolate 1), SA (*S. aureus* ATCC 25923), and *E. coli* (*E. coli* DH5α ATCC) were grown in Luria-Bertani medium at 37°c. STM Δ*invC* and STM Δ*fliC* were a kind gift from Michael Hensel (University of Osnabrueck, Germany).

### Cell Culture

The human intestinal epithelial cell line (Caco-2, NCCS, Pune) and human cervix adenocarcinoma cell line (HeLa, a kind gift from Prof. Anjali Karandae) were maintained in DMEM (Sigma–Aldrich) containing 10% FBS (Fetal Bovine serum, Gibco) at 37°C in 5% CO_2_. For polarizing Caco-2 cells, cells were seeded in permeable tissue culture (0.45 μTranswell^R^ Corning) inserts for 7 days, until they form tight monolayer. For screening of PAMPs that can induce BPI expression in Caco-2 cells, 10^6^ cells were seeded in 6 well plate. Twenty-four hours post-seeding, cells were treated with STM LPS 100 ng/mL (Sigma–Aldrich) and STM Flagellin 500 ng/mL, HK STM (Heat killed STM) or were infected with STM, STY, or SA at an MOI of 10 as explained below. Cells were treated with 100 nM ATLA4 (aspirin-triggered lipoxin A4, Calbiochem), and was used as a positive control. To check the importance of pore-forming toxins and ion efflux in inducing BPI expression, cells were treated with Streptolysin O (SLO, Sigma–Aldrich) for 24 h, Listeriolysin O 1 μg (LLO, Sigma–Aldrich) for 24 h, Nigericin 6 μM (Sigma–Aldrich) and Thapsigargin 500 nm (Sigma–Aldrich) for 30 min followed by media change. p38 MAPK inhibitor, SB203580 20 nM (Calbiochem) was added 1 h before addition of Nigericin, SLO or SA infection. For all experiments using Nigericin and ATLA4, DMSO (Sigma–Aldrich) was used as a solvent control. To check the role of osmotic stress on BPI expression, cells were treated with 1% cellulose (Sigma–Aldrich) along with SA infection. Cells were processed for either confocal microscopy or western blot as explained below. Flagellin was isolated from STM as previously described (Marathe et al., 2016).

### Animal Model

To check BPI expression in infected mice, BALB/c and C3H/Hej (TLR4 deficient) female mice aged 6 weeks were infected intragastrically with 10^8^ bacteria per ml (STM, SA, STM Δ*invC*, and STM Δ*fliC*). For infection with *S. flexneri*, mice were infected intraperitoneally with 10^8^ bacteria per ml of SHG as described by Yang et al. (2014). All mice were euthanized 2 days post-infection. The intestine was isolated, Small intestine was divided into three equal parts and was considered as duodenum, jejunum, and ileum and was processed for RNA isolation or histopathological analysis as described below. To quantify bacterial invasion in the intestine of infected mice, the intestine was homogenized in tissue homogenizer in 1 ml PBS. The homogenate was plated at different dilutions in *Salmonella Shigella* agar. CFU was calculated and normalized to per gram weight of intestine. For evaluating colitis in infected mice, colon length was calculated as the length between the rectum and distal colon. Mice were maintained in SPF conditions throughout the experiment.

### Bacterial Infection and Proliferation Assay in Cell Lines

Cells were infected with SA, STM or STY at a MOI of 10. Extracellular bacteria were removed 30 min post-infection and cells were maintained in 100 μg/ml Gentamicin for 1 h to kill any extracellular bacteria. Infected cells were maintained in DMEM containing 10 μg/ml of Gentamicin.

For checking the effect of BPI knockdown on STM survival, Caco-2 cells were transfected with BPI dsRNA or scrambled dsRNA control. Twenty-four hours post-treatment, cells were treated with SLO (2U) or Nigericin (6 μM) for 24 h. Cells were infected with STM as described above. For quantifying bacterial survival, Caco-2 cells were lysed using 0.1% TritonX-100 (Sigma–Aldrich) 2 h post-infection. The Bacterial number in cell lysates were quantified by plating the cell lysates in *Salmonella Shigella* agar. Percentage survival was calculated with respect to untreated control. Percentage survival was calculated as follows.

$$\begin{array}{c} \%\mathrm{Survival}\;=\;\mathrm{CFU}\;(\mathrm{Treated})/CFU\;(\mathrm{untreated})\;\times \;100 \end{array}$$

### Knockdown of BPI in Caco-2 Cells

In order to knockdown BPI in Caco-2 cells, BPI dsRNA was designed against three regions within the gene a) GGAGCTGAAGAGGATCAAGATTCCTGACTACTCAGACAGCTTTAAGATCAAGCATCTTGGGAAGGGGCATTATAGCTTCTACAGCATGGACATCCGTGAATTCCAGCTTCCCAGTTCCCAGATAAGCATGGTGCCCAATGTGGGCCTTAAGTTCTCCATCAGCAACGCCAATATCAAGATC b) GTGTCCACGTGC ACATCTCAAAGAGCAAAGTCGGGTGGCTGATCCAACTCTTCCACAAAAAAATTGAGTCTGCGCTTCGAAACAAGATGAACAGCCAGGTCTGCGAGAAAGTGACCAATTCTGTATCCTCCAAGCTGCAACCTTATTTCCAGACTCTGC c) GGGTCTTGAAGATGACCCTTAGAGATGACATGATTCCAAAGGAGTCCAAATTTCGACTGACA ACCAAGTTCTTTGGAACCTTCCTACCTGAGGTGGCCAAGAAGTTTCCCAACATGAAGATACAGATCCATGTCTCAGCCTCCACC. All dsRNA were obtained from chromous biotech. Transfection was done using Oligofectamine as recommended by the manufacturer (Invitrogen, Life Technologies). Transfection was done for 24 h in DMEM without FBS. Post-transfection, cells were maintained in DMEM with 10% FBS.

### Real-Time PCR Analysis

RNA was isolated from Caco-2 cells using TRIzol reagent (Invitrogen) as per manufacturer’s protocol. For RNA isolation from the intestine, the intestine was homogenized in bead beater in 1 ml TRIzol reagent. After DNase treatment 2 μg of RNA was used for cDNA synthesis using Tetra reverse transcriptase (Bioline). QRT PCR was performed using the Kapa SYBR Green RT-PCR kit (Kapa Biosystems) as per manufactures protocol in an Applied Biosystems^®^ ViiA7^TM^ Real-time PCR instrument. The following primers were used for detecting BPI level by real-time PCR. hBPI forward primer, 5′ATGAACAGCCAGGTCT3′, hBPI reverse primer, 5′GGTCATTACTGGCAG3′. Human actin forward primer, 5′GGTGGCTTTTAGGATGGCAAG3′, Human actin reverse primer 5′ACTGGAACGGTGAAGGTGACAG3′. Mouse BPI forward primer 5′GGTAAGAAGGAAAACAAATGCC3′, Mouse BPI reverse primer 5′AACCACCTGCTGCCAA3′. Mouse actin forward primer 5′CAGCAAGCAGCAGTACGATG3′, Mouse actin reverse primer 5′GCAGCTCAGTAACAGTCCG3′. The following primers were used to detect IL-8 expression by real time PCR. Human IL-8 forward primer 5′ATGACTTCCAAGCTGGCCGTG3′, Human IL-8 reverse primer 5′TTATGAATTCTCAGCCCTCTTCAAAAACTTCTC3′. Expression levels were quantified with respect to actin (internal control) using the 2^-ΔΔct^ method.

### Western Blot

10^6^ Caco-2 cells were treated with indicated reagents or were infected with bacteria. Cells were lysed at indicated time points using RIPA buffer [50 mM Tris–HCl (pH 7.4), 1% NP-40, 0.25% Sodium deoxycholate, 150 mM NaCl, 1 mM EDTA, 1 mM PMSF, 1 μg/ml of each aprotinin, leupeptin, pepstatin, 1 mM Na_3_VO_4_ and 1 mM NaF] containing 10% Protease inhibitor cocktail (Sigma–Aldrich). Protein obtained from cell lysates were quantified using Bradford assay and 80 μg of total protein was loaded on 10% SDS–PAGE. Proteins were resolved and transferred to PVDF membrane (Millipore). Blocking was done using 4% Bovine serum albumin (BSA, Sigma–Aldrich) in TBST (20 mM Tris–HCl (pH 7.4), 137 mM NaCl, and 0.1% Tween 20) for 1 h to prevent non-specific binding. The blots were incubated in anti-BPI antibody (Sigma–Aldrich) overnight at 4°C followed by anti-rabbit HRP (DSHB, The University of Lowa) for 2 h. Antibodies were reconstituted in TBST solution containing 5% BSA. For quantifying β-Actin, blots were incubated with anti-β-Actin antibody conjugated with HRP (Sigma–Aldrich). Immunoblots were visualized by enhanced chemiluminescence reagent (Biovision) as per manufacturer’s instructions. For probing proteins in the same region of PVDF membrane, blots were incubated in stripping buffer [62.5 mM Tris–HCl (pH 6.8), 2% SDS and 0.7% β-mercaptoethanol] at 60°C on a shaker. Blots were scanned, BPI bands were quantified with respect to corresponding β-actin bands using the Multi Gauge software (FUJIFILM). Representative images were shown in illustrations.

### Confocal Microscopy

To visualize BPI expression in Caco-2 cells, cells were seeded on glass coverslips or permeable tissue culture inserts before treatment or infection. Post-treatment cells were washed with PBS and fixed with 3.5% paraformaldehyde for 15 min. Cells were permeabilized using 1% Saponin dissolved in PBS with 3% BSA. Immunostaining was done using anti-BPI antibody (Sigma–Aldrich) followed by anti-rabbit Alexa 647 antibody (DSHB, University of Iowa). To visualize TLR4 expression in Caco-2 cells, cells were stained with the anti-FLAG antibody (Sigma–Aldrich) followed by anti-rabbit Alexa 488 antibody (DSHB, University of Iowa). For experiments using *S. aureus* infection, coverslips were blocked with anti-human IgG (Sigma–Aldrich) for 2 h prior to addition of primary antibody to inhibit non-specific interaction of protein-A and anti-BPI antibody. Preliminary results confirm that the signal is specific to BPI and not a cross reacting signal from Protein-A present in *S. aureus*. Image acquisition was done with a Zeiss confocal microscope (LSM Meta 710). Mean fluorescent intensity of each image were quantified using Zen Blue Software.

### TLR4 Overexpression in Caco-2 Cells

Caco-2 cells were transfected with pCMV: hTLR4-FLAG (a kind gift from Dr. Markus Schnare, University of Marburg, Germany) using PEI transfection reagent (Sigma–Aldrich). Twenty-four hours post-transfection, cells were washed with sterile PBS and were maintained in DMEM containing 10% FBS. Transfected cells were examined by confocal microscopy for expression of membrane FLAG-tagged human TLR4.

### Histological Analysis of Intestine

BALB/c mice were dissected 2 days post-infection in order to evaluate the pathological effects. Intestines were fixed overnight in 4% paraformaldehyde and then dehydrated and embedded in paraffin. Paraffin-embedded intestine (ileum) was sectioned using a microtome. Intestinal sections (5 μm) were processed for hematoxylin and eosin staining to identify nuclear and protein (cytoplasmic material). In a double blinded scoring, randomly selected intestinal sections were scored independently by two experts (Dr. Ritu Gambher Chadha and Dr. Joan Maria Vynetta). Histological scores were given based on the criteria explained by Rath et al. (1996).

### Statistical Analysis

The data were subjected to statistical analysis by applying Student’s *t*-test by using Graph Pad prism 4 software.

## Author Contributions

AB and DC conceived the study. AB performed the Experiments. AB and DC analyzed the data and wrote the manuscript.

## Conflict of Interest Statement

The authors declare that the research was conducted in the absence of any commercial or financial relationships that could be construed as a potential conflict of interest.
