# Supplementary material for: Epithelial Cell Damage Activates Bactericidal/Permeability Increasing-Protein (BPI) Expression in Intestinal Epithelium
Source: Front Microbiol. 2017 Aug 15;8:1567. doi: 10.3389/fmicb.2017.01567 (PMC5559428; doi:10.3389/fmicb.2017.01567)
Supplement: Supplementary file 1 [file Presentation_1.PDF]

Supplementary figures

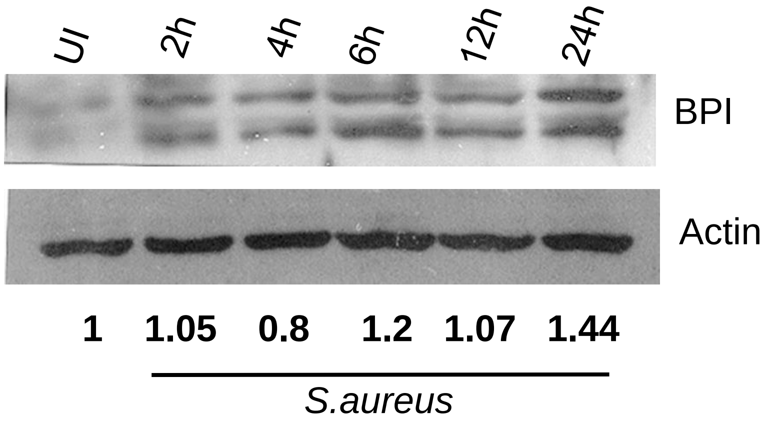

FIGURE S1 . HeLa cells were infected with SA for indicated time points. BPI expression in cell lysates were quantified by Western Blot. (n=3 experiments).

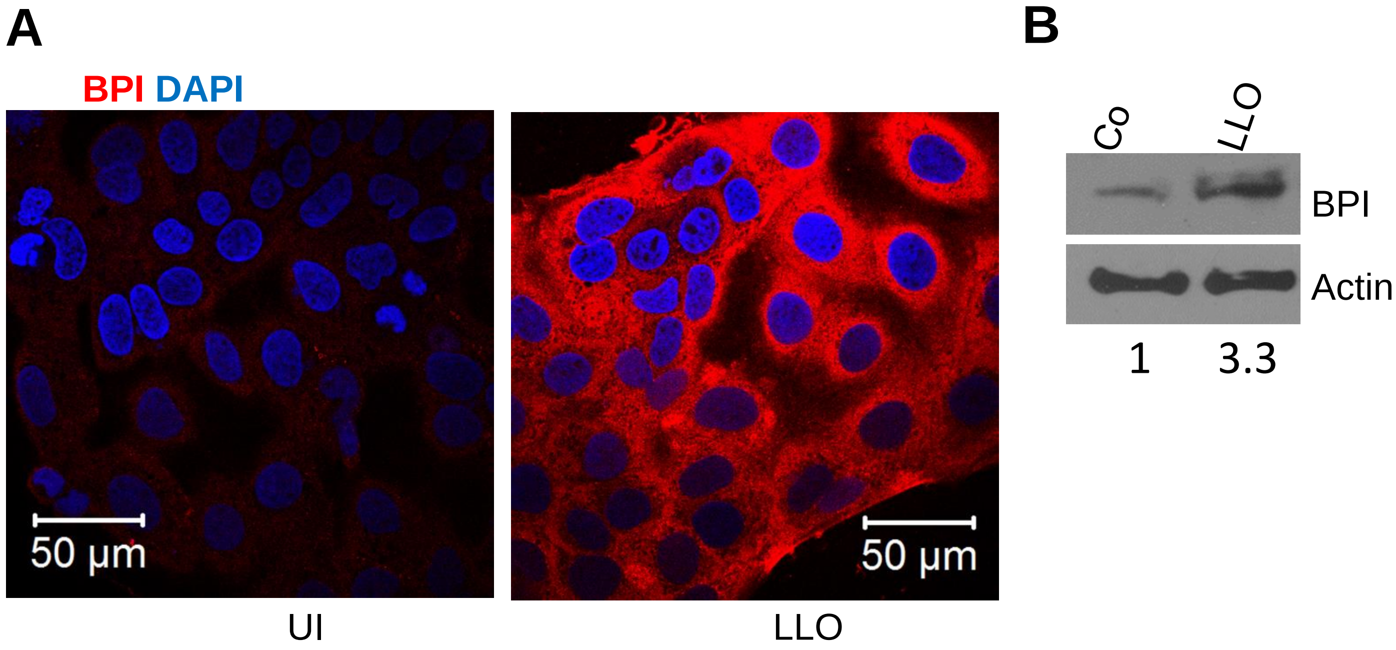

FIGURE S2. Caco2 cells were treated with Listeriolysin (LLO, 1µg/mL).**A)** Cells were fixed 24h post treatment and were immunostained with anti BPI antibody followed by anti-antibody conjugated with Alexa 647 (red). Nuclei were labelled with 4′,6-diamidino-2-phenylindole (DAPI) (blue). Cells were imaged by confocal microscopy. Representative images are shown. **B)** Cells were lysed 24h post treatment and BPI expression was quantified by western blot.

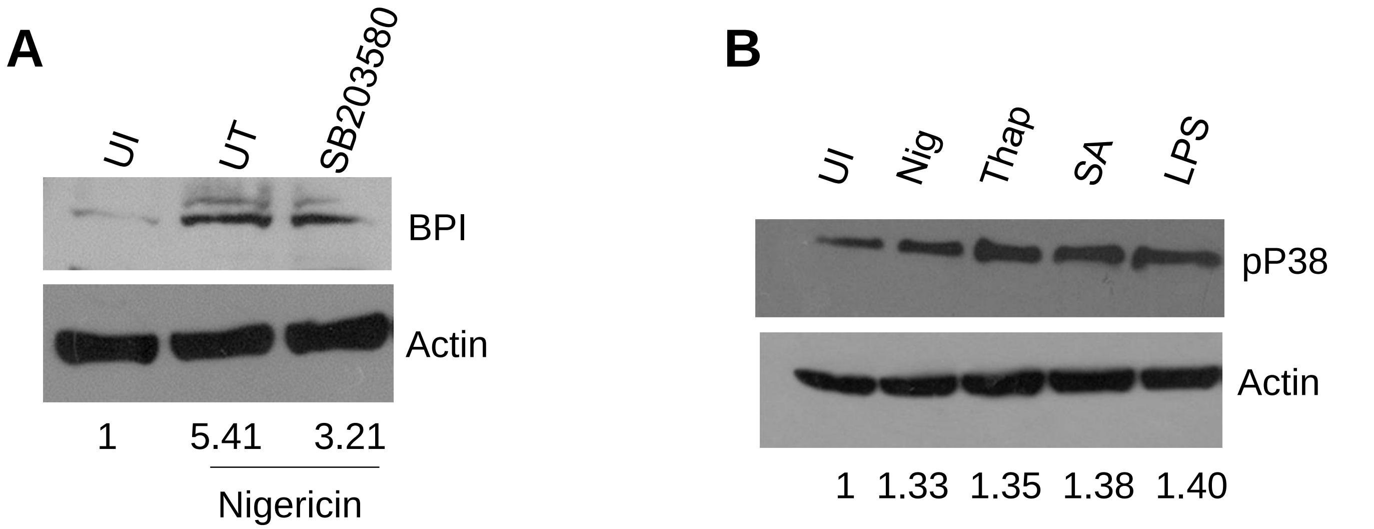

FIGURE S3. A) HeLa cells were either left untreated or treated with SB203580 1h before Nigericin (6µm) treatment. Cells were lysed 24h post treatment and BPI levels were quantified by western blot. **B)** Cac02 Cells were treated with Thapsigargin (500nm) ,Nigericin (6µm),SA (MOI 10) or LPS (100ng/mL). 24h post treatment, cells were lysed and pP38 levels were quantified using western blot.

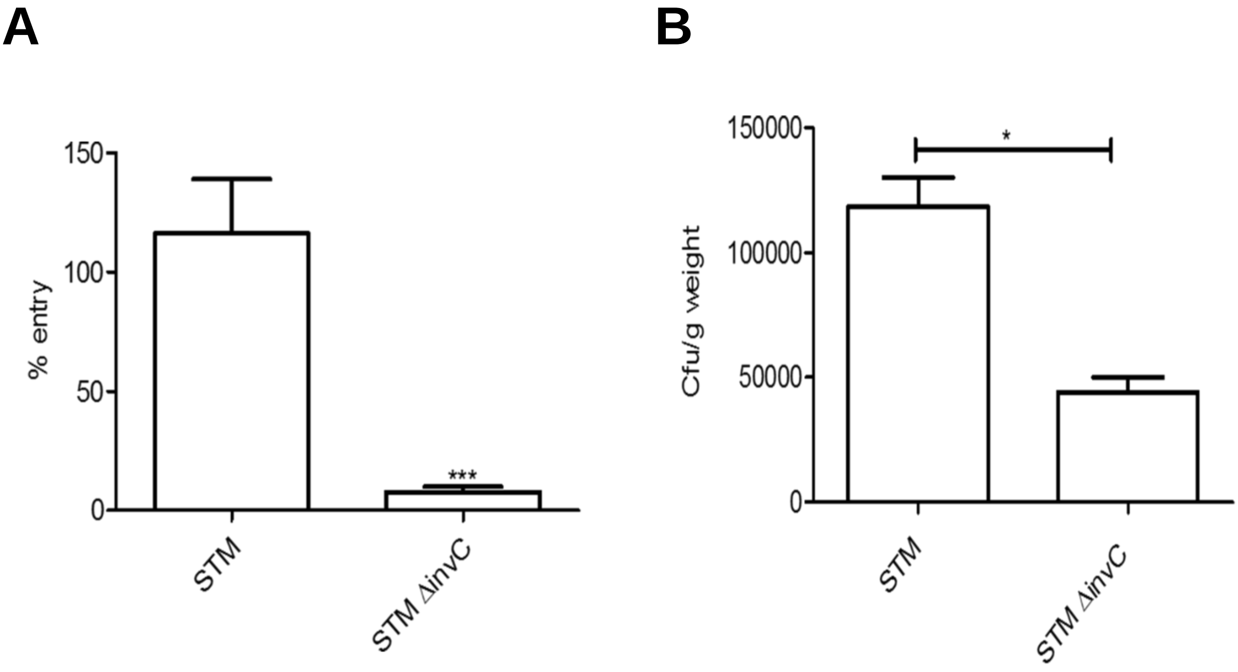

FIGURE S4. A) Caco2 cells were infected with STM or STM *ΔinvC*. Percentage entry was calculated by plating the cell lysates 1h post infection. **B)** Mice were infected intragastrically with 10^8 bacteria, 2 days post infection small intestine was isolated and bacterial numbers were determined by homogenizing and plating the homogenate in Salmonella Shigella agar.

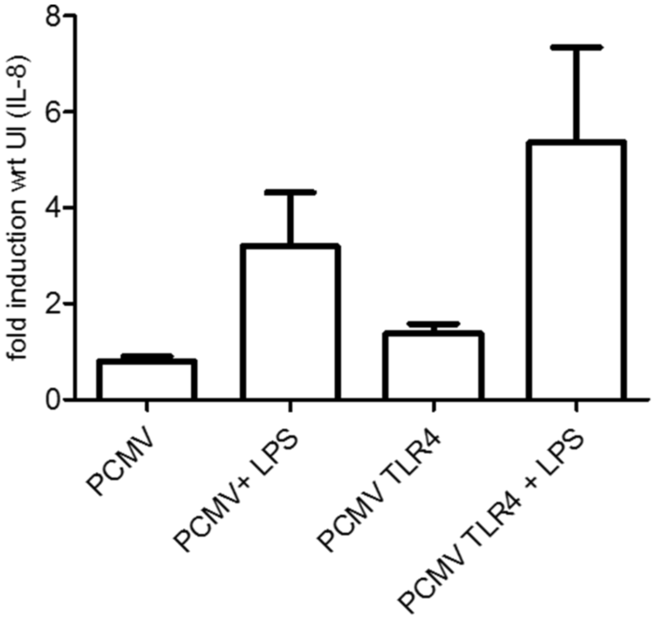

FIGURE S5. Caco2 cells transfected with PCMV or PCMV TLR4 was exposed to LPS for 2h.Total RNA was isolated and IL-8 expression was quantified by real time PCR. (n=3 experiments)

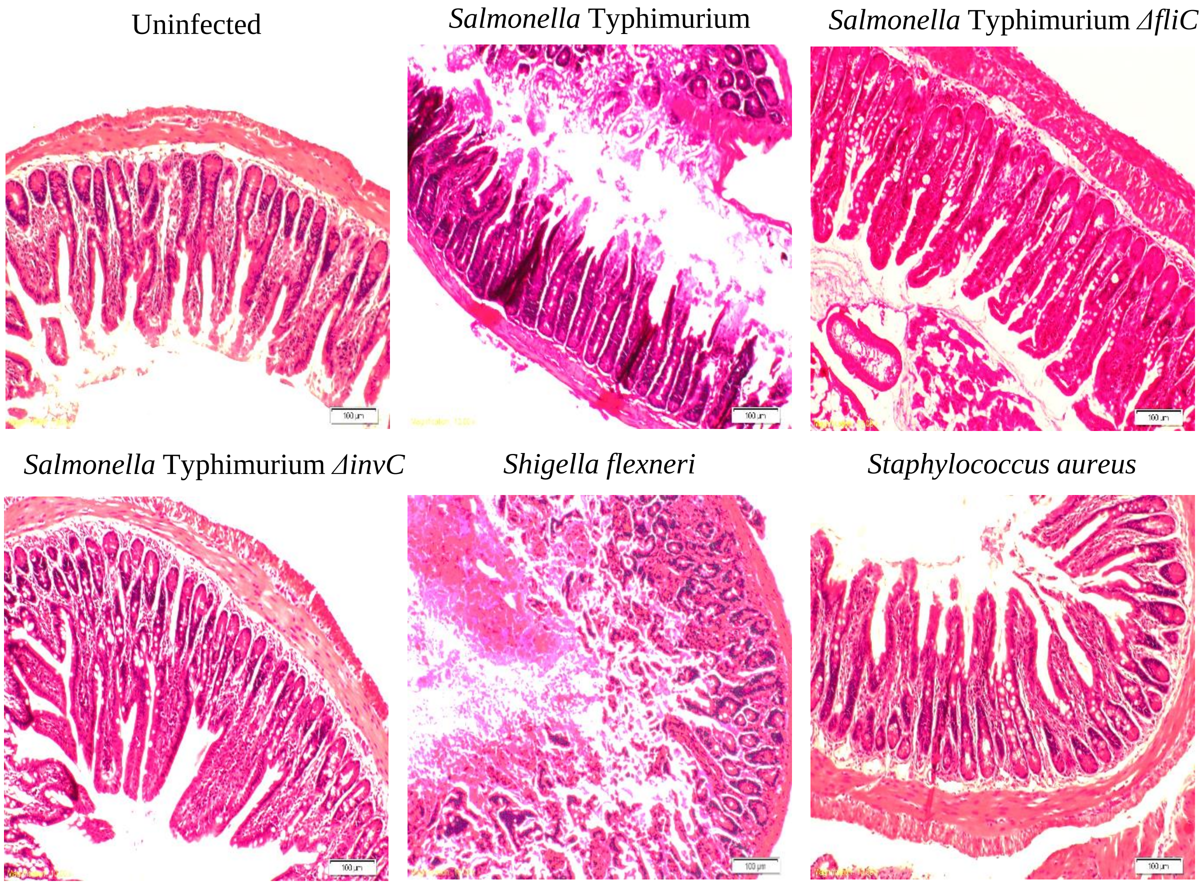

**Criterion**	UI	STM	STM Δ*fliC*	STM Δ*invC*	SHG	SA
Inflammatory Cells	**0**	**2**	**1**	**1**	**3**	**1**
Goblet Cells	**0**	**1**	**1**	**1**	**3**	**0**
Mucosa thickening	**0**	**0**	**0**	**0**	**1**	**0**
Submucosa cell infiltration	**0**	**2**	**0**	**0**	**2**	**1**
Destruction of architecture	**0**	**1**	**0**	**0**	**3**	**0**

FIGURE S6. Histology of ileal inflammation in Balb/c mice 2 days post-infection with indicated bacteria (representative images are shown). Below: Histological score to quantify the degree of intestinal inflammation in infected mice.
